# Sediments: sink, archive, and source of contaminants

**DOI:** 10.1007/s11356-022-24041-1

**Published:** 2022-11-10

**Authors:** Aurea C. Chiaia-Hernández, Carmen Casado-Martinez, Pablo Lara-Martin, Thomas D. Bucheli

**Affiliations:** 1 Institute of Geography & Oeschger Centre for Climate Change Research, University of Bern, Bern, Switzerland; 2 Ecotox Center, 1015 Lausanne, Switzerland; 3 Physical Chemistry Department, Faculty of Marine and Environmental Sciences, Campus of International Excellence of the Sea (CEI·MAR), University of Cadiz, 11510 Puerto Real, Spain; 4 Environmental Analytics, Zurich, Switzerland

Sediments are sources and sinks of contaminants and play an important role in mediating pollutants across environmental compartments of terrestrial and aquatic ecosystems. In surface waters (lakes, slowly flowing or dammed rivers, estuaries, oceans), organic and inorganic contaminants are either dissolved or sorbed to suspended matter and sediment particles according to their chemical properties. In the case of strong sorption, settling of suspended particles and sediment formation scavenge contaminants out of the water phase, resulting in the accumulation of contaminants in the beds of rivers and lakes.

Under anoxic conditions, contaminants are less biodegradable; therefore, preservation of pollutants in anaerobic sediments (e.g., sediment beds and lake sediments) create a long-lasting potential source as has recently been demonstrated in streams, lake, and marine sediments (Chiaia-Hernández et al. 2017, 2020b; Lara-Martin et al. 2006; Lara-Martín et al. 2015). Once in the sediment, contaminants may transform, accumulate or recycle, and interact with water above, with the groundwater, and with biota living in and above the sediment.

Sediments are very complex dynamic systems that are affected by hydrodynamic factors (storms, subaquatic slumps, bioturbation by dwelling organisms), physicochemical processes (sorption, redox reactions), and microbial transformations. Therefore, a correct evaluation of these processes is of fundamental importance for environmental risk assessment and the prediction of the long-term fate of chemical pollution in sediments. Chronic contaminant inputs to sediments at trace levels occur very widely from regional to global scales by means of riverine, run-off, industrial activities, and atmospheric input pathways. Additionally, sediments can also be severely contaminated either continuously (harbors, waste discharges, naval activities) or by accidents (oil spills, fires). Compared to established standards for water, air and to some extent soil quality, the ecotoxicological thresholds for polluted sediments are less well defined in national and international legislation. Therefore, advancing the interdisciplinary science of contaminated sediments with chemicals released into the environment directly (e.g., effluents from wastewater treatment plants) or indirectly (e.g., the runoff of pesticides applied on agricultural land, drift of sprayed pesticides, and accidental spillage or atmospheric deposition of toxins) is of high societal relevance.

Currently available methods for the determination of chemicals at trace levels provide very valuable information with results useful for pollutant source identification and apportionment recognition and historic pollution reconstruction. For example, gas chromatography or liquid chromatography–mass spectrometry (GC/LC–MS), using single- or triple-quadrupole instrumentation has been shown to be selective, very sensitive, and fast. Moreover, advances in analytical techniques such as high-resolution mass spectrometry (HRMS) have led to new opportunities in environmental trace analysis (Gago-Ferrero et al. 2018; Krauss et al. 2010). The coupling of HRMS with LC through electrospray ionization or with GC through electron impact or chemical ionization and the use of MS/MS capabilities have produced reliable screening data for molecular ions, product ions, and their MS/MS fragments (Krauss et al. 2010). The capabilities of HRMS have been demonstrated in different publications in recent years with the detection of thousands of organic contaminant and transformation products (known and unknown) with and without reference standards.

Today, given the available analytical instrumentation, a wide range of contaminants can be studied. For example, compounds such as polychlorinated dibenzo-*p*-dioxins and -furans and polychlorinated biphenyls (PCBs) can be used as markers of atmospheric deposition in sediments from remote water bodies (e.g., alpine lakes), (Bogdal et al. 2008, 2009; Iozza et al. 2008; Zennegg et al. 2007) or as secondary source of primarily legacy hazardous substances to the water column from sediments in urban and industrial harbors, bays, and rivers (Assefa et al. 2014; Mustajärvi et al. 2017; Sobek et al. 2015). Likewise, surfactants (e.g., linear alkylbenzene, linear alkylbenzene sulfonate, and nonylphenol) and their degradation intermediates have been detected in sediment cores, showing that these compounds are still presently detectable in the cores and can be markers of sewage pollution (Lara-Martin et al. 2006). Moreover, pesticides, pharmaceuticals, and personal care products, compounds thought not to be present in sediments, have been detected in lakes impacted with agricultural inputs and urban settings (Chiaia-Hernandez et al. 2014; Chiaia-Hernández et al. 2020a, 2020b; Lara-Martín et al. 2015; Löffler et al. 2005, Löffler and Ternes 2003). Let us not forget about heavy metals and trace element that have recorded contamination from industrial activities and other anthropogenic activity (Hsu et al. 2016; Magesh et al. 2011). Furthermore, sediment records have helped to decipher the impact of climate change by using biogeochemical molecules, inorganic markers, stable isotopes, and natural forcings, to name a few (Jiskra et al. 2022; Wanner et al. 2008; Zander et al. 2021).

So far, the research on sediment analysis with dated and/or undisturbed varved sediments has pointed to three main findings. Sediments (i) provide a comprehensive view of known and unknown contamination since the onset of industrialization, (ii) systematically document the time when contaminants appear and disappear, and (iii) explore their changes over time as a response to different management practices and regulatory interventions or environmental changes. In addition, spatial analysis of sediments can give insights on the transport and deposition of contaminants.

Environmental risk assessments of contaminants in sediments are performed for supporting decision-making in very different contexts and situations. These include prospective and generic risk assessments conducted for (pre)marketing authorizations of chemicals, pesticides, pharmaceuticals, etc. as well as retrospective assessments, e.g., as diagnosis tools in the identification of ecological effects and the responsible stressors at contaminated areas, or to monitor effectiveness of remediation. In pre-marketing authorizations, risk assessment for the sediment compartment is mainly triggered by the results of partitioning and persistence assessments, which are performed according to standard protocols to estimate the partitioning coefficient n-octanol/water, and targeting transformation in aquatic sediment systems for persistence assessment (OECD 1995, 2002a, b, 2004). Since the introduction of existing protocols and guidelines for assessing partitioning and persistence, which date back to the early 00 s, several shortcomings have been identified. For example, approaches for assessing partitioning are challenged when dealing with substances at the boundaries of traditional models such as ionizable organic compounds (ECETOC 2013, Sigmund et al. 2022) or substances of unknown or variable composition, complex reaction products, or biological material (Whale et al. 2021). Degradation studies used for persistence assessment have also shown high variability and lack of reproducibility due to numerous factors such as the formation of non-extractable residues, the type of sediment used in the experiments, or the amount and composition of degrader biomass (Hughes et al. 2020; Seller et al. 2020; Whale et al. 2021). If key triggers for regulatory sediment risk assessment are challenged, it is therefore not surprising that recent measurement campaigns are reporting increasing concentrations in sediments for substances traditionally thought of as non-sorptive and therefore not traditionally considered in sediment monitoring (Chiaia-Hernández et al. 2020b; Fairbairn et al. 2015; Joerss et al. 2019; Lara-Martín et al. 2015) or substances that have been out of the radar such as non-traditional polycyclic aromatic hydrocarbons (An et al. 2020).

Overall, the refinement and update of existing protocols and regulatory guidelines triggering risk assessment for the sediment compartment are warranted. Recently, bioavailability has been started to be considered within risk assessment for a more realistic assessment rather than relying on the traditional approach of using total-extractable concentrations (Ortega-Calvo et al. 2015). In addition, proposals for the standardization of protocols for assessing non-extractable residues (Loeffler et al. 2020) and for a better consideration of abiotic and biotic controls in transformation studies (Seller et al. 2020) should improve persistence evaluations in chemical risk assessment (Schäffer et al. 2018; Whale et al. 2021). Nevertheless, results of measurement campaigns showing increasing concentrations in sediments can also trigger sediment risk assessment, e.g., in the WFD (EC 2018), and should also trigger re-assessments of substances already on the market. The development of analytical capacities and existing international data repositories such as the Network of reference laboratories, research centers, and related organizations for monitoring of emerging environmental substances (NORMAN) and the upcoming European Partnership for the Assessment of Risk from Chemicals (PARC) offers a unique opportunity for feedback loops between monitoring activities and different regulatory frameworks.

Nowadays, sediments need to be investigated not just by environmental scientists but using an interdisciplinary approach which involve (i) earth scientists (sedimentologists) who play a pivotal role for identifying ideal sampling sites, analyzing sedimentation regimes, and dating of the sediment layers; (ii) analytical chemists who provide qualitative and quantitative determinations at trace to ultra-trace concentrations (10–6 to 10–9 g per kg sediment); (iii) microbiologists who evaluate the impact of aerobic and anaerobic biotransformation processes occurring in the sediments; and (iv) ecotoxicologists who assess the potential impact of contaminated sediment on aquatic ecosystems and associated risks for human exposure. Merging recent scientific advances in these different disciplines is required to achieve significant progress and overcome current challenges in environmental risk assessments of contaminated sediments.

In 2015, the first International Conference of Contaminated Sediments, ContaSed 2015, was held at the Swiss Federal Institutes of Technology (ETH) Conference Center Stefano Franscini, Monte Verita, Ascona, Switzerland (Bogdal et al. 2016). The aim of ContaSed 2015 was to provide a platform for experts as well as junior researchers from different scientific disciplines to present recent results and exchange about novel approaches on the analysis, effects, risk assessment, and remediation of contaminated sediments. Building on the momentum from ContaSed 2015, a second ContaSed was organized for June 2020 and later postponed to 2021 due to the outbreak of the coronavirus disease (COVID-19). ContaSed 2021 was held online from the 9th to the 11th of June 2021 at the University of Bern. ContaSed 2021 consolidated a multidisciplinary and multilevel meeting for exchanging and discussing on the most recent trends in contaminated sediments research and practice. ContaSed2021 gathered more than 150 different internationally leading experts and junior researchers from 32 countries. A total of 40 live oral presentations and 50 pre-recorded poster presentations from experts in environmental chemistry, sedimentology, ecotoxicology, limnology, and ecology, to name a few, contributed to the scientific knowledge on sources, transport, sinks, cycling, and effects of legacy and emerging organic pollutants, heavy metals, microplastics, and engineered nanomaterials with different topics distributed in five different session. ContaSed 2021 was offered completely free of charge thanks to the infrastructure and support provided by the Oeschger Centre for Climate Change Research (OCCR) and the University of Bern. Due to the interest in sediment research, the lack of platforms for scientific exchange on sediment research, and the over well interest on the conference, a follow-up conference (ContaSed 2025) every 5 years is planned.

This special issue contains a collection of different articles, in which the authors present diverse work in sediment research from heavy metals to legacy and perfluorinated compounds. We hope that by getting out some of the latest sediment research, we can raise awareness of this very important matrix within the environmental science community and the broader scientific community interested in environmental issues.

## References

[CR1] An Y, Hong S, Yoon SJ, Cha J, Shin KH, Khim JS (2020). Current contamination status of traditional and emerging persistent toxic substances in the sediments of Ulsan Bay. South Korea Mar Pollut Bull.

[CR2] Assefa AT, Sobek A, Sundqvist KL, Cato I, Jonsson P, Tysklind M, Wiberg K (2014). Temporal trends of PCDD/Fs in Baltic Sea sediment cores covering the 20th century. Environ Sci Technol.

[CR3] Bogdal C, Schmid P, Kohler M, Müller CE, Iozza S, Bucheli TD, Scheringer M, Hungerbühler K (2008). Sediment record and atmospheric deposition of brominated flame retardants and organochlorine compounds in Lake Thun, Switzerland: lessons from the past and evaluation of the present. Environ Sci Technol.

[CR4] Bogdal C, Schmid P, Zennegg M, Anselmetti FS, Scheringer M, Hungerbühler K (2009) Blast from the past: melting glaciers as a relevant source for persistent organic pollutants. Environ Sci Technol 43:(21), 8173–8177. 10.1021/es901628x 10.1021/es901628x 19924940

[CR5] Bogdal C, Chiaia-Hernández AC, Giger W (2016). Recent sediments: environmental chemistry, ecotoxicology and engineering. Environ Sci Pollut Res.

[CR6] Chiaia-Hernandez A, Schymanski E, Kumar P, Singer H, Hollender J (2014). Suspect and nontarget screening approaches to identify organic contaminant records in lake sediments. Anal Bioanal Chem.

[CR7] Chiaia-Hernández AC, Günthardt BF, Frey MP, Hollender J (2017). Unravelling contaminants in the anthropocene using statistical analysis of liquid chromatography–high-resolution mass spectrometry nontarget screening data recorded in lake sediments. Environ Sci Technol.

[CR8] Chiaia-Hernández AC, Scheringer M, Müller A, Stieger G, Wächter D, Keller A, Pintado Herrera MG, Lara-Martin PA, Bucheli TD, Hollender J (2020a) Target and suspect screening analysis reveals persistent emerging organic contaminants in soils and sediments. Sci Total Environ 140181 10.1016/j.scitotenv.2020.140181 32927551

[CR9] Chiaia-Hernández AC, Zander P, Schneider T, Szidat S, Lloren R, Grosjean M (2020b) A high-resolution historical record of plant protection products deposition documented by target and nontarget trend analysis in a Swiss lake under anthropogenic pressure. Environ Sci Technol 10.1021/acs.est.0c04842 32940459

[CR10] EC (2018) European Commission. 2018. Technical guidance for deriving environmental quality standards, Guidance Document No 27 under the Common Implementation Strategy for the Water Framework Directive (2000/60/EC). Technical Report 2011–055. Updated version 2018. Document endorsed by EU Water Durectors at their meeting in Sofia on 11–12 June 2018. Brussels (BE): European Commission

[CR11] ECETOC (2013) Environmental exposure assessment of ionisable organic compounds. Technical Report 123 – Environmental risk assessment of ionisable compounds. ISSN-2079–1526–123 (online)

[CR12] Fairbairn DJ, Karpuzcu ME, Arnold WA, Barber BL, Kaufenberg EF, Koskinen WC, Novak PJ, Rice PJ, Swackhamer DL (2015). Sediment-water distribution of contaminants of emerging concern in a mixed use watershed. Sci Total Environ.

[CR13] Gago-Ferrero P, Krettek A, Fischer S, Wiberg K, Ahrens L (2018). Suspect screening and regulatory databases: a powerful combination to identify emerging micropollutants. Environ Sci Technol.

[CR14] Hsu L-C, Huang C-Y, Chuang Y-H, Chen H-W, Chan Y-T, Teah HY, Chen T-Y, Chang C-F, Liu Y-T, Tzou Y-M (2016). Accumulation of heavy metals and trace elements in fluvial sediments received effluents from traditional and semiconductor industries. Sci Rep.

[CR15] Hughes CB, Brown DM, Camenzuli L, Redman AD, Arey JS, Vione D, Wang N, Vaiopoulou E (2020). Can a chemical be both readily biodegradable AND very persistent (vP)? Weight-of-evidence determination demonstrates that phenanthrene is not persistent in the environment. Environ Sci Eur.

[CR16] Iozza S, Muller CE, Schmid P, Bogdal C, Oehme M (2008). Historical profiles of chlorinated paraffins and polychlorinated biphenyls in a dated sediment core from Lake Thun (Switzerland). Environ Sci Technol.

[CR17] Jiskra M, Guédron S, Tolu J, Fritz SC, Baker PA, Sonke JE (2022). Climatic controls on a holocene mercury stable isotope sediment record of Lake Titicaca. ACS Earth Space Chem.

[CR18] Joerss H, Apel C, Ebinghaus R (2019). Emerging per- and polyfluoroalkyl substances (PFASs) in surface water and sediment of the North and Baltic Seas. Sci Total Environ.

[CR19] Krauss M, Singer H, Hollender J (2010). LC–high resolution MS in environmental analysis: from target screening to the identification of unknowns. Anal Bioanal Chem.

[CR20] Lara-Martin PA, Petrovic M, Gomez-Parra A, Barcelo D, Gonzalez-Mazo E (2006). Presence of surfactants and their degradation intermediates in sediment cores and grabs from the Cadiz Bay area. Environ Pollut.

[CR21] Lara-Martín PA, Renfro AA, Cochran JK, Brownawell BJ (2015). Geochronologies of Pharmaceuticals in a sewage-impacted estuarine urban setting (Jamaica Bay, New York). Environ Sci Technol.

[CR22] Löffler D, Ternes TA (2003). Determination of acidic pharmaceuticals, antibiotics and ivermectin in river sediment using liquid chromatography-tandem mass spectrometry. J Chromatogr A.

[CR23] Löffler D, Rombke J, Meller M, Ternes TA (2005). Environmental fate of pharmaceuticals in water/sediment systems. Environ Sci Technol.

[CR24] Loeffler D, Hatz A, Albrecht D, Fligg M, Hogeback J, Ternes TA (2020). Determination of non-extractable residues in soils: towards a standardised approach. Environ Pollut.

[CR25] Magesh NS, Chandrasekar N, Vetha Roy D (2011). Spatial analysis of trace element contamination in sediments of Tamiraparani estuary, southeast coast of India. Estuar Coast Shelf Sci.

[CR26] Mustajärvi L, Eek E, Cornelissen G, Eriksson-Wiklund A-K, Undeman E, Sobek A (2017). In situ benthic flow-through chambers to determine sediment-to-water fluxes of legacy hydrophobic organic contaminants. Environ Pollut.

[CR27] NORMAN Substance Database – NORMAN SusDat. https://www.norman-network.com/nds/susdat/. Accesed on 17.07.2019

[CR28] OECD (1995) Test No. 107: Partition coefficient (n-octanol/water): Shake Flask Method . 10.1787/9789264069626-en

[CR29] OECD (2004) Test No. 117: Partition coefficient (n-octanol/water), HPLC method. OECD Guidelines for the Testing of Chemicals, Section 1: Physical-Chemical properties. 10.1787/9789264069824-en

[CR30] OECD (2002a) Test No. 308: Aerobic and anaerobic transformation in aquatic sediment systems. OECD Guidelines for the Testing of Chemicals, Section 3: Environmental fate and behavior. 10.1787/9789264070523-en.

[CR31] OECD (2002b) Test No. 308: Aerobic and anaerobic transformation in aquatic sediment systems. 10.1787/9789264070523-en

[CR32] Ortega-Calvo J-J, Harmsen J, Parsons JR, Semple KT, Aitken MD, Ajao C, Eadsforth C, Galay-Burgos M, Naidu R, Oliver R, Peijnenburg WJGM, Römbke J, Streck G, Versonnen B (2015). From bioavailability science to regulation of organic chemicals. Environ Sci Technol.

[CR33] Schäffer A, Kästner M, Trapp S (2018). A unified approach for including non-extractable residues (NER) of chemicals and pesticides in the assessment of persistence. Environ Sci Eur.

[CR34] Seller C, Honti M, Singer H, Fenner K (2020). Biotransformation of chemicals in water–sediment suspensions: influencing factors and implications for persistence assessment. Environ Sci Technol Lett.

[CR35] Sigmund G, Arp HPH, Aumeier BM, Bucheli TD, Chefetz B, Chen W, Droge STJ, Endo S, Escher BI, Hale SE, Hofmann T, Pignatello J, Reemtsma T, Schmidt TC, Schönsee CD, Scheringer M (2022). Sorption and mobility of charged organic compounds: how to confront and overcome limitations in their assessment. Environ Sci Technol.

[CR36] Sobek A, Sundqvist KL, Assefa AT, Wiberg K (2015). Baltic Sea sediment records: unlikely near-future declines in PCBs and HCB. Sci Total Environ.

[CR37] Wanner H, Beer J, Bütikofer J, Crowley TJ, Cubasch U, Flückiger J, Goosse H, Grosjean M, Joos F, Kaplan JO, Küttel M, Müller SA, Prentice IC, Solomina O, Stocker TF, Tarasov P, Wagner M, Widmann M (2008). Mid- to Late Holocene climate change: an overview. Quatern Sci Rev.

[CR38] Whale G, Parsons J, van Ginkel K, Davenport R, Vaiopoulou E, Fenner K, Schaeffer A (2021). Improving our understanding of the environmental persistence of chemicals. Integr Environ Assess Manag.

[CR39] Zander PD, Żarczyński M, Tylmann W, Rainford S, Grosjean M (2021). Seasonal climate signals preserved in biochemical varves: insights from novel high-resolution sediment scanning techniques. Clim past.

[CR40] Zennegg M, Kohler M, Hartmann PC, Sturm M, Gujer E, Schmid P, Gerecke AC, Heeb NV, Kohler H-PE, Giger W (2007). The historical record of PCB and PCDD/F deposition at Greifensee, a lake of the Swiss plateau, between 1848 and 1999. Chemosphere.

